# Broadband Sound Administration Improves Sleep Onset Latency in Healthy Subjects in a Model of Transient Insomnia

**DOI:** 10.3389/fneur.2017.00718

**Published:** 2017-12-21

**Authors:** Ludovico Messineo, Luigi Taranto-Montemurro, Scott A. Sands, Melania D. Oliveira Marques, Ali Azabarzin, David Andrew Wellman

**Affiliations:** ^1^Division of Sleep and Circadian Disorders, Department of Medicine, Brigham and Women’s Hospital, Harvard Medical School, Boston, MA, United States; ^2^Division of Sleep and Circadian Disorders, Department of Neurology, Brigham and Women’s Hospital, Harvard Medical School, Boston, MA, United States; ^3^Respiratory Medicine and Sleep Laboratory, Department of Experimental and Clinical Sciences, University of Brescia, Spedali Civili di Brescia, Brescia, Italy; ^4^Department of Allergy, Immunology and Respiratory Medicine, Central Clinical School, The Alfred and Monash University, Melbourne, VIC, Australia; ^5^Pulmonary Division, Heart Institute (InCor), Hospital das Clínicas, University of São Paulo School of Medicine, São Paulo, Brazil

**Keywords:** filtered white noise, sleep onset latency, insomnia alternative treatments, effective sleep aids, increased arousal threshold

## Abstract

**Background:**

Insomnia is a major public health problem in western countries. Previous small pilot studies showed that the administration of constant white noise can improve sleep quality, increase acoustic arousal threshold, and reduce sleep onset latency. In this randomized controlled trial, we tested the effect of surrounding broadband sound administration on sleep onset latency, sleep architecture, and subjective sleep quality in healthy subjects.

**Methods:**

Eighteen healthy subjects were studied with two overnight sleep studies approximately one week apart. They were exposed in random order to normal environmental noise (40.1 [1.3] dB) or to broadband sound administration uniformly distributed in the room by two speakers (46.0 [0.9] dB). To model transient insomnia, subjects went to bed (“lights out”) 90 min before usual bedtime.

**Results:**

Broadband sound administration reduced sleep onset latency to stage 2 sleep (time from lights out to first epoch of non-rapid eye movement-sleep stage 2) (19 [16] vs. 13 [23] min, *p* = 0.011; median reduction 38% baseline). In a subgroup reporting trouble initiating sleep at home (Pittsburgh Sleep Quality Index section 2 score ≥ 1), sound administration improved subjective sleep quality (*p* = 0.037) and the frequency of arousals from sleep (*p* = 0.03).

**Conclusion:**

In an experimental model of transient insomnia in young healthy individuals, broadband sound administration significantly reduced sleep onset latency by 38% compared to normal environmental noise. These findings suggest that broadband sound administration might be helpful to minimize insomnia symptoms in selected individuals.

## Statement of Significance

Despite the lack of data regarding the efficacy of broadband sound administration on sleep onset latency and sleep depth in a quiet environment, many people use noise conditioners in their rooms as makeshift sleep aids. The current study is the first examining a large population of healthy subjects (undergoing a model of transient insomnia) with complete polysomnographies and indicates that broadband sound administration significantly reduces sleep onset latency to stage 2. Moreover, it shows that the “sleep latency” component of the Pittsburgh Sleep Quality Index (PSQI) can be used as a clinical predictor of a better response to the broadband sound administration.

## Introduction

Insufficient and low-quality sleep is a major public health problem that has been linked to motor vehicle crashes, industrial disasters, and medical and other occupational errors (1). Insomnia symptoms affect 35% of the population worldwide (2). Although transient insomnia, by definition, lasts for a short time and is difficult to predict, it can be assessed clinically with questionnaires such as the Insomnia Severity Index (3) and the PSQI (4). Previous research has shown transient insomnia is related to many psychiatric and medical consequences, as well as chronic insomnia, and eventually results in an increased economic burden due to healthcare costs (1) and all-cause mortality risk (5). Hence, treatment is crucial.

Although many drug therapies have already been developed for insomnia, their risk-benefit profiles need to be accurately balanced in every patient. Pharmacological therapy is often secondary to psychological-behavioral approaches (6, 7), but the number of people primarily using sleep-inducing drugs to increase or improve sleep is steadily increasing, despite the side effects of these therapies, such as addiction and rebound reactions (8).

As a non-pharmaceutical alternative and, most of all, with technological advancements, evidence is accumulating in support of a role for broadband sound administration to mask environmental noise disturbances. Noise disturbances from sources either inside or outside the home are common sources of problems with sleep initiation and maintenance (9–11). Despite the lack of well-designed, randomized controlled trials, a National Sleep Foundation survey from 2012 reported that 5% of the American population already uses sound conditioners such as fans, air purifiers, vacuum cleaners, broadband (e.g., “white”) noise devices in their rooms as makeshift sleep aids (12).

Broadband sound administration is based on the “auditory masking phenomenon” (13) whereby a masker (e.g., constant low-level noise) decreases the audibility of a disturbing signal (e.g., the indoor/outdoor noise). Masking occurs because the difference between the loudness of the background sound level and the disturbing signal is reduced, leading to a reduction in the bilateral auditory evoked response to the signal and an increase in the subjective arousal threshold (14, 15).

Moreover, sound administration may also affect sleep directly through the impact of the acoustic stimulus on brain electrical activity (16). Broadband sound administration—involving the addition of a background sound comprising a broad range of frequencies (e.g., bass, mid, treble)—have been used in several pilot studies and small trials. Stanchina et al. (17) performed polysomnography (PSG) in four subjects under baseline conditions as well as during a simulated night in an intensive care unit (ICU) with and without white noise. They demonstrated that both the arousal index at baseline and during white noise administration were significantly inferior as compared to the night with simulated ICU noise exposure. Similarly, a recent study (18) showed better scores in the PSQI in 30 ICU patients exposed to broadband sound as compared to another 30 patients who were not. However, the effect of broadband sound in the environmental low noise setting remains unclear.

In an attempt to find new, alternative solutions to increase sleep quality, we aimed to assess the effect of broadband sound administration on sleep onset latency and sleep architecture.

Differently than previous research, in order to achieve consistent findings, we used complete, attended PSG in a relatively large population sample.

## Materials and Methods

### Subjects

This controlled, randomized, single-blinded crossover trial was performed at the Brigham and Women’s Hospital and Harvard Medical School, Boston, MA, USA. Twenty healthy subjects aged 20–65 years were recruited between August 2016 and February 2017. Every individual underwent a history and physical examination to ensure eligibility for the study. Exclusion criteria were insomnia or other sleep-related disorders (including sleep deprivation and circadian diseases) and acute or chronic usage of any medication known to influence sleep or arousal state. All the individuals were asked to maintain the same sleep schedules throughout the study and to abstain from consuming alcoholic or caffeinated beverages 24 and 12 h before the study, respectively. For all the subjects, anthropometric data, PSQI (4), and Epworth sleepiness scale (ESS) (19) were recorded before the study.

### Trial Registration

The protocol was approved by the Partners Institutional Review Board at Brigham and Women’s Hospital. All subjects provided written informed consent before enrollment in the study. The trial was registered on http://www.clinicaltrials.gov (NCT02945254).

### Measurements

The following data were acquired from the overnight sleep studies: electroencephalography (EEG), electrooculography (EOG), chin and leg electromyography, electrocardiography, thoracic and abdominal movements, body position, and oxyhemoglobin saturation (Nonin Medical, Plymouth, MN, USA). Respiration was recorded through a nasal cannula attached to a pressure transducer (Validyne Engineering, Northridge, CA, USA) referenced to atmosphere.

### Protocol

Two overnight sleep studies were performed on weekdays approximately one week apart: a control night (ambient noise) and a treatment night (broadband sound administration or “sound blanket”). Broadband sound was administered *via* a purpose-built stereo speaker system (Nightingale^®^ Cambridge Sound Management, Waltham, MA, USA), placed on two opposite walls in the room, at the right and left sides of the bed. The two speakers provided constant filtered noise at a higher sound pressure than the indoor environmental noise (46 [0.9] versus 40.1 [1.3] dB, respectively, *p* < 0.001). The sound level of 46 dB was chosen because it is at the mid-range of values usually taken into account for masking environmental noise due, for example, to road traffic (20), and it is similar to what the World Health Organization defines as the maximum noise pressure compatible with a good night’s sleep (21). The specific frequencies of sound were specially matched to the room with the goal of making it unclear where the source of the sound was located. The volume of sound administered was controlled with a customized mobile application (Cambridge Sound Management). Sound pressure levels were measured with a smartphone application (SPL meter, Studio Six Digital, Seattle, WA, USA).

A model of transient insomnia was used in which subjects went to bed (“lights out”) 90 min before their usual bedtime. Many in-lab models to simulate transient insomnia have been proposed (22–25). Although advancing the sleep phase by a variable number of hours is an accepted way to induce transient insomnia, it has not been validated to our knowledge. We decided to adopt a 90-min phase advance because that is the average time for a normal sleep cycle to be completed (26). At the end of both nights, the subjective sleep quality was assessed with a visual analog scale (VAS) (27), and the level of morning alertness was evaluated by the Stanford sleepiness scale (SSS) (28).

Randomization was performed by dedicated software (Randomizer for clinical trial, Medsharing, France). For each night, the subjects arrived at the sleep laboratory at approximately 7:00 p.m. and were given the same sleep opportunity.

### Data Analysis

Sleep stages, arousals, apneas, and hypopneas were scored by a certified sleep technician blinded to the treatment allocation using American Academy of Sleep Medicine (AASM) standard criteria (29).

Our primary measure of sleep onset latency was the interval (in minutes) between lights out and stage 2 sleep (N2), chosen to guarantee a certain degree of sleep consolidation, generally eliminating the possibility of transient sleep onsets. We also recorded sleep latency to stage 1 sleep (N1), defined as either the time to the start of the first three consecutive epochs of N1, or an epoch of any other stage, whatever is earliest (30). Latency to stage 3 sleep (N3) and rapid eye movement (REM) sleep were also measured.

Furthermore, according to the AASM definitions (29), the following parameters were analyzed: total sleep time (TST), time in bed (TIB), sleep efficiency (SE), total recording time, and latency to N1, N2, N3, and REM sleep, wake after sleep onset time (WASO), apnea-hypopnea index (AHI), arousal index (ArI), and average sleep oxyhemoglobin saturation (SaO_2_ mean).

### Statistics

A two-tailed, Wilcoxon test was used to compare paired data groups, and a Mann–Whitney rank sum test was used to compare unpaired data groups. To assess the relative strength of factors that might influence the subjective improvement in sleep quality, two stepwise multiple regression analyses were performed with the difference in the VAS scores between the nights as the dependent variable. In the first, we included only objectively measured independent variables such as improvement in sleep efficiency between the nights; difference in total time of N1, N2, N3, and REM sleep between the nights; difference in TST; difference in WASO; difference in ArI; sleep onset latency to N2; latency of the first three epochs of N1. In the second multivariable regression, the independent variables examined were the subjective parameters: usual sleep onset latency; PSQI global score; PSQI component two (sleep latency) and five (sleep disturbances). The variables selected for the multivariate model were those passing a univariate test threshold (*p* < 0.25), as suggested by Bursac et al. (31).

Data are expressed as the mean ± SD or the median (interquartile range), and statistical significance was accepted if *p* < 0.05. Statistical analyses were performed using Graph Pad Prism 6.0 (Graph Pad Software, La Jolla, CA, USA) and SPSS 19.00 (IBM, Armonk, NY, USA).

## Results

**Figure 1:**
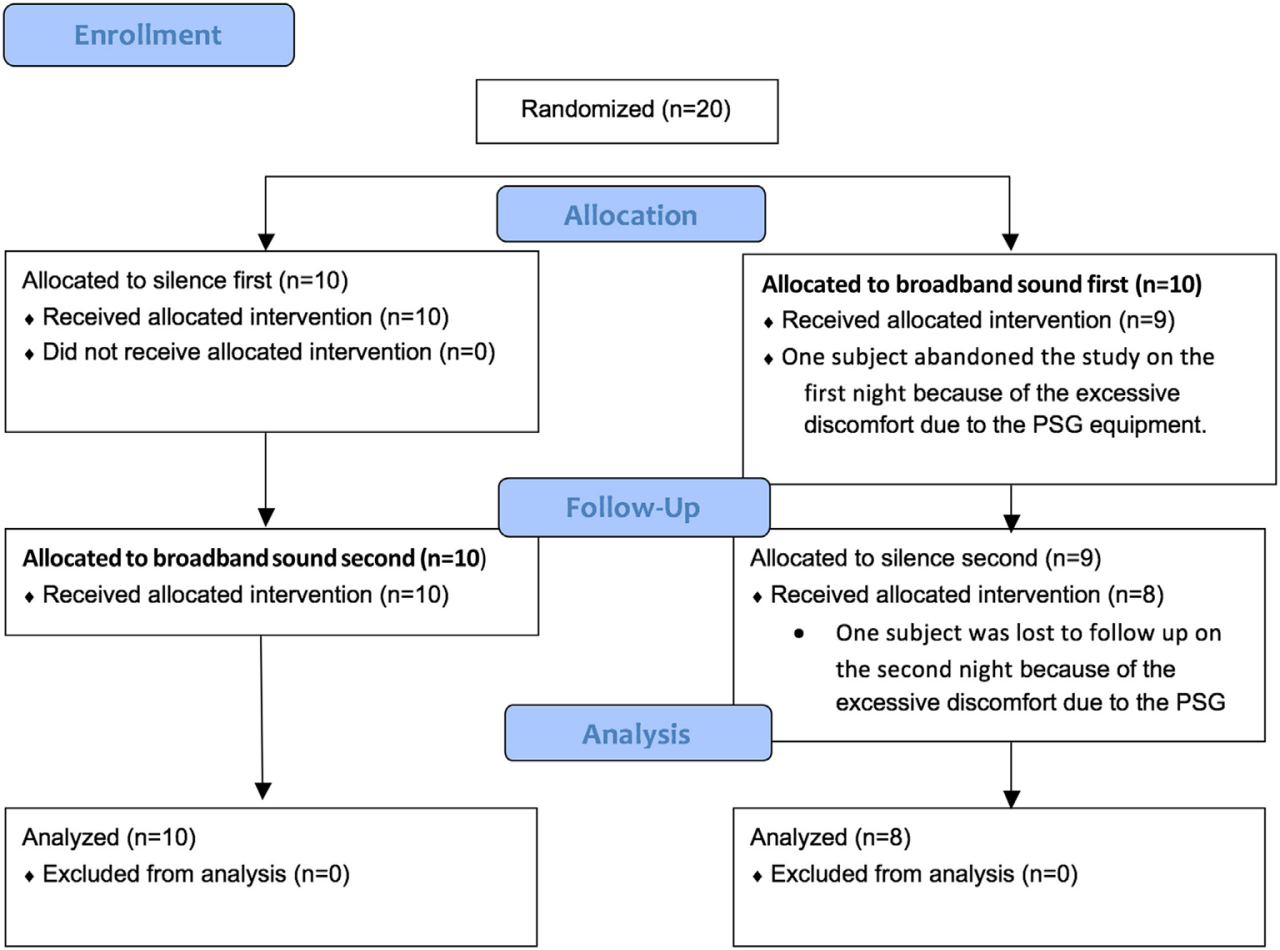
Diagram of the clinical trial.

A total of 20 subjects were enrolled in the study; two subjects dropped out due to equipment intolerance and thus data from 18 subjects were analyzed. A diagram of the clinical trial is represented in Figure 1. Subjects’ anthropometric and baseline data are summarized in Table 1. Subjects were, on average, normal weight. Usual sleep duration, bedtime and sleep onset latency refer to the month prior to the study. None of the participants were current smokers (two subjects stated that they quit smoking for a period longer than 1 month prior to the study).

**Table 1:** Anthropometric and baseline data for all the subjects of the study.

Age (years)	28.5 [13.5]
Female gender, *n* (%)	9 [50]
BMI (kg/m^2^)	24.2 [3.8]
PSQI, global score	3 [2.5]
ESS, score	5 [3.75]
Usual sleep duration (h)	7.4 [0.94]
Usual bedtime, h:min (min)	12:00 [60]
Usual sleep onset latency (subjective) (min)	17.5 [20]

*Data are represented as median [interquartile range]*.

*BMI, body mass index; PSQI, Pittsburgh Sleep Quality Index; ESS, Epworth Sleepiness Scale*.

### Main Findings

**Figure 2:**
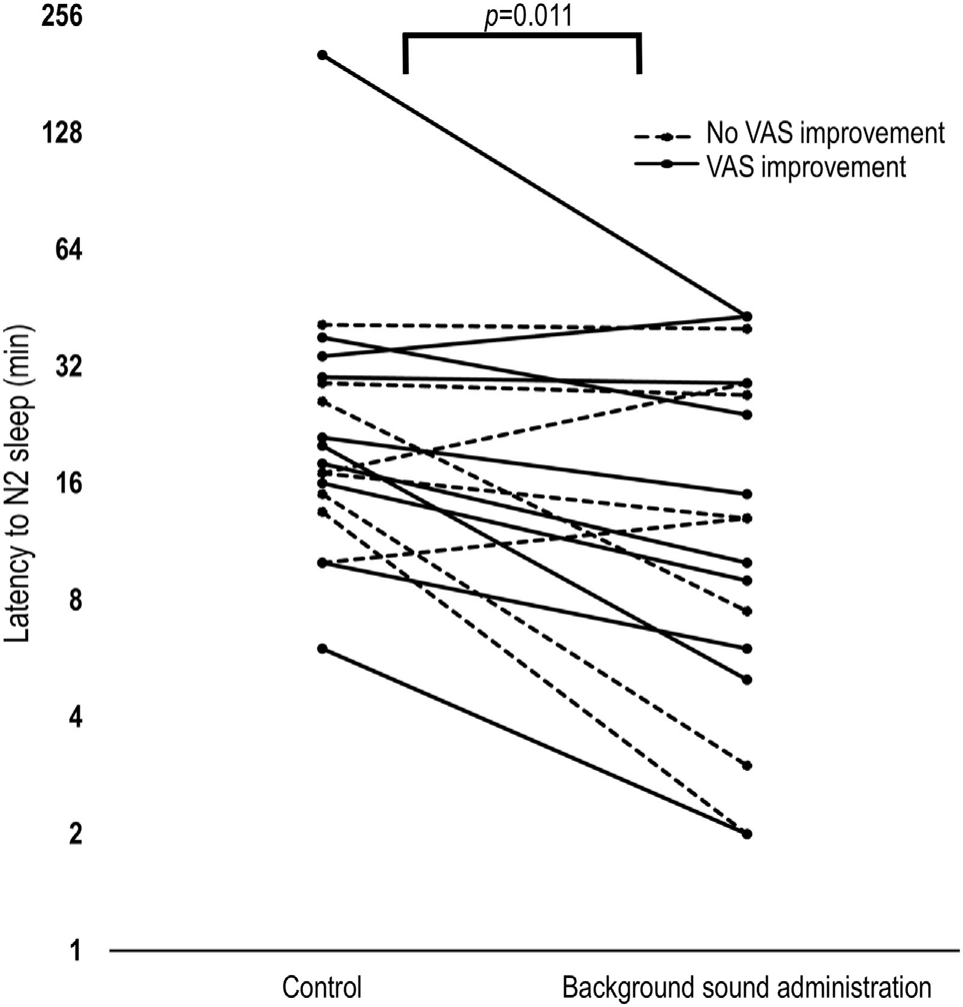
Latency to stage 2 sleep modification between nights. Background sound administration significantly reduced N2 latency by 38% as compared to the control night. The subjects who subjectively improved on the treatment night [change in VAS > 0] exhibited a significant median reduction (42%) of N2 sleep latency, while the others did not. Note that the *y*-axis is presented in logarithmic scale. VAS, visual analog scale; N2, non-rapid-eye movement stage 2.

**Figure 3:**
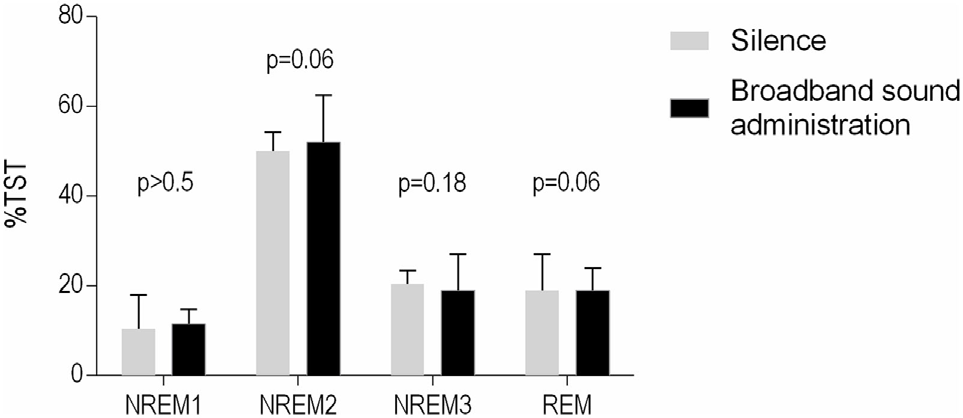
Subjects’ sleep architecture from both nights. Data are expressed as median [interquartile range]. The administration of broadband sound increased NREM1 sleep but had no significant effect on the other sleep stages.

The sleep onset latency to stable stage N2 sleep was significantly diminished by administering broadband sound [median reduction in sleep onset latency was 38% (66), *p* = 0.011, Figure 2]. We also assessed the effect of study order (second versus first night) and observed no significant additional effect on N2 latency of study order (trend = −4.8 ± 2.7 min, *p* = 0.084; mixed model analysis). Adjusting for study order did not diminish the observed effect of treatment (−6.8 ± 2.7 min, *p* = 0.018; mixed model analysis). There was no reduction in the latency to stage N1, N3, or REM sleep (Table 2) and no change in the sleep architecture (Table 3; Figure 3) between nights. However, a trend for a slight overall increase in N2 [median increase of 6 (11)% of TST, *p* = 0.065] and reduction in REM sleep [median reduction −3 (−4)% of TST, *p* = 0.059] was observed when the sound blanket was used (Figure 3). Data regarding subjective sleep quality are shown in Table 2: no significant differences were found between nights as a whole.

**Table 2:** Subjects’ data on sleep stage latencies and on subjective sleep quality from both nights.

	Control	Broadband sound night	*p*
Stage 1 latency (min)	13.75 [13.5]	8.5 [22.5]	0.155
Stage 2 latency (min)	19 [16.27]	13 [23.25]	0.011
Stage 3 latency (min)	31.5 [15.5]	26.5 [40]	0.758
REM latency (min)	109 [91.05]	120 [90.5]	0.558
VAS, total score	7 [2.5]	7 [1.5]	0.132
SSS, total score	2.5 [1]	2 [2]	0.535

*Data are expressed as median [interquartile range]*.

*REM, rapid eye movement sleep; VAS, visual analog scale; SSS, Stanford sleepiness scale*.

**Table 3:** Subjects’ sleep parameters from both nights.

	Control	Broadband sound night	*p*
TST (min)	454 [116.2]	443 [164]	0.648
SE, %TIB	88 [11.5]	87.5 [12.75]	0.989
WASO (min)	39 [21]	36 [56.25]	0.475
ArI (events/h)	15.45 [14.65]	13.85 [8.65]	0.17
AHI (events/h)	1.9 [5]	1.2 [3.15]	0.247
SaO_2_ mean (%)	99 [0.6]	99 [0.92]	0.236

*Data are expressed as median [interquartile range]*.

*SE, sleep efficiency; WASO, wake after sleep onset; ArI, arousal index; AHI, apnea hypopnea index; SaO_2_ mean, oxyhemoglobin saturation mean value during sleep*.

### Factors Explaining Improvement in Subjective Sleep Quality

**Figure 4:**
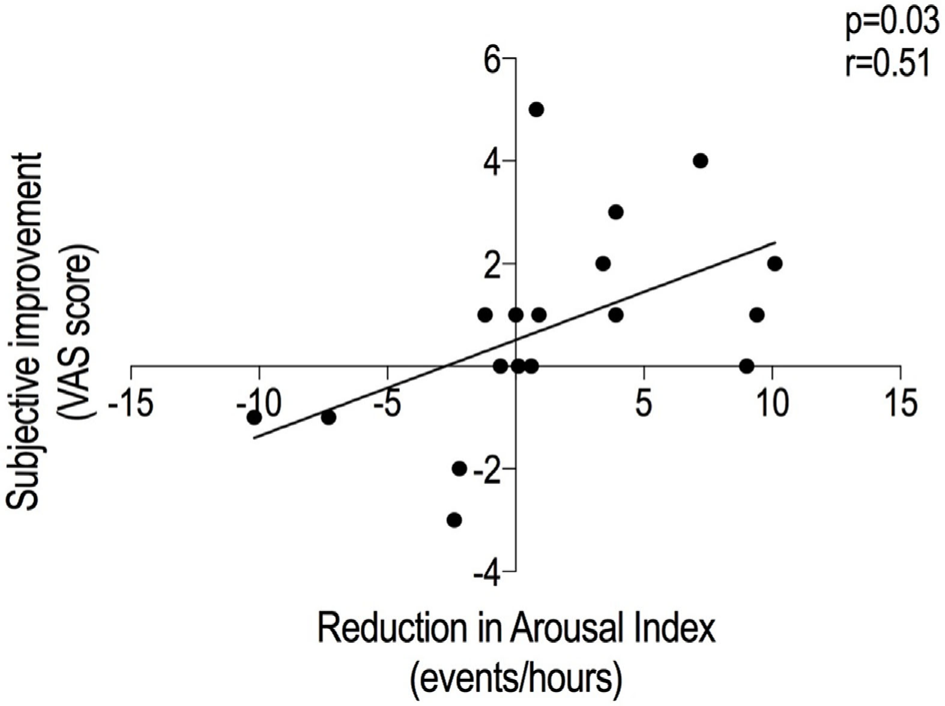
Factors explaining the improvement in the subjective sleep quality. The VAS improvement resulted to have a direct relationship with the arousal index reduction between nights. The diminished number of arousals on the noise arm could have improved the subjective quality of sleep on the same night. VAS, visual analog scale.

The subjects who subjectively improved on the treatment night (change in VAS > 0, *n* = 10) exhibited a significant reduction of N2 sleep latency [20.5 (10.5) vs. 12.5 (26.8) min, *p* = 0.033]. By contrast, the others (*n* = 8) did not [17 (14.4) vs. 13 (24.4) min, *p* = 0.266]. Multivariate analysis revealed that the strongest correlate of the improvement in subjective sleep quality was the reduction in ArI with treatment (*r* = 0.51, *p* = 0.03) (Figure 4).

### Subgroup Analysis

A separate multivariate analysis also showed that the sleep latency score on the PSQI was the only predictive determinant of improved subjective sleep quality (*r* = 0.495, *p* = 0.03). Thus, we divided subjects into two groups (*post hoc*) based on their PSQI sleep latency score; “faster sleepers” (*n* = 5) were defined as subjects with a score of 0, and “slower sleepers” (*n* = 13) were defined by those with a score ≥ 1 (usual sleep latency > 15 min, or at least one episode of transient insomnia in the previous month, i.e., “cannot get to sleep within 30 min”). Treatment in the “slower sleepers” significantly improved subjective sleep quality (VAS) (*p* = 0.037) and lowered the arousal index [15 (13.55) vs. 13.6 (8.7) min, *p* = 0.03], with a trend for a slight improvement of latency to N2 [18 (10.5) vs. 15 (27), *p* = 0.0688]. “Faster sleepers” did not exhibit these improvements.

## Discussion

The main finding of this study was that the exposure to broadband sound administration (Nightingale^®^ sound blanket) reduced the onset to stable stage N2 sleep by 38% in 18 healthy subjects undergoing an experimental model of transient insomnia. While subjective sleep quality did not improve as a group, a subgroup of subjects (“slower sleepers” who reported higher sleep onset latencies at home, based on PSQI sleep latency score) exhibited subjective and objective sleep improvements with intervention.

### Physiological Mechanisms

Two potential explanations for these results include: (1) the auditory properties of the sound treatment reduced the sensitivity to the 40 dB environmental noise and allowed subjects to initiate sleep faster, with individual sensibility or (2) stochastic resonance in the noise signal enhanced neural synchronization in the brain during sleep (16, 32). In fact, it has previously been shown that noise treatment lowers EEG signal complexity (i.e., fractal component of EEG) and increases brain wave synchronization (16). This effect was attributed to stochastic resonance, a statistical non-linear phenomenon which allows a non-zero, optimal level of noise to increase the detection of weak stimuli or enhance the information content of a signal (i.e., trains of action potentials or signals generated by a neuronal assembly) (33). It has been demonstrated that neural synchronization is facilitated by the addition of optimal amounts of random fluctuations, or “noise,” to a neural network, whereas less than optimal amounts have less effect and larger than optimal amounts disrupt synchronization. Stochastic resonance is then dose-dependent up to a threshold (34). Therefore, we can speculate that the sound pressure level we used might have been just high enough to favor an initial neural synchronization as an anticipated sleep onset, but not high enough to significantly prolong deep sleep stages.

### Clinical Implications

Our findings have important clinical implications; the effect size of filtered white noise in this group of healthy subjects was similar to the effect of eszopiclone 2 mg, a non-benzodiazepine hypnotic widely prescribed in the United States and showed to reduce median sleep latency to consolidated sleep by 5 min as compared to placebo (23). Due to the risks of tolerance and dependence with hypnotics, cognitive-behavioral therapy is often the preferred choice of treatment for insomnia (35). Therefore, a non-pharmacologic aid with an efficacy similar to an approved hypnotic could provide patients with a safer alternative. We speculate that auditory masking may be effective in patients with insomnia. Interestingly, evidence suggests that anxiety and tiredness—commonly associated with insomnia (2)—may in fact increase sensitivity to auditory disturbances (36). In such individuals, auditory masking might be particularly effective as an intervention. Further research is warranted. Finally, it is worthwhile mentioning that our results indicate a shortening to stage N2 (i.e., consolidated) sleep rather than simply a reduction in the latency to transient (stage N1) sleep.

To our knowledge, this is the largest clinical trial to date testing the effect of sound therapy on sleep quality using a complete, attended PSG. One previous study testing white noise on sleep architecture was performed by Terzano et al. (37) in 12 patients. The authors found that sleep microstructure was negatively impacted by white noise at a sound pressure of 45 dB. However, the ambient sound pressure in this experiment never exceeded 27 dB, which suggests that a higher sound pressure differential (and, possibly, a higher perceived noise intensity) might have undesirable effects on sleep. Another potential difference was that, in our protocol, the filtered noise was homogeneously diffused in the room (not localized), and it had been customized with specific features to fit the room acoustics. Spencer and collaborators’ work (38) was the most recent among many studies trying to use white noise as a sleep aid. They studied a small cohort of neonates with a sound pressure ranging from 67 to 72.5 dB and found that 80% of neonates fell asleep within 5 min in response to noise, compared with only 25% of controls who fell asleep spontaneously. However, the lack of standard measurements for sleep architecture or quality makes the results difficult to interpret and reproduce. Williamson (39) administered white noise for 3 days to 30 patients recovering from coronary bypass surgery and found that their subjective sleep quality, assessed by a VAS, improved as compared to 30 matched controls. However, again no objective sleep parameters were measured in this study. Finally, Scott (40) evaluated eight subjects for eight consecutive nights in the sleep laboratory under approximately 93 dB white noise or quiet conditions. The author showed that, during the noise night, N1 and N2 time were increased, while REM time was decreased.

**Figure 5:**
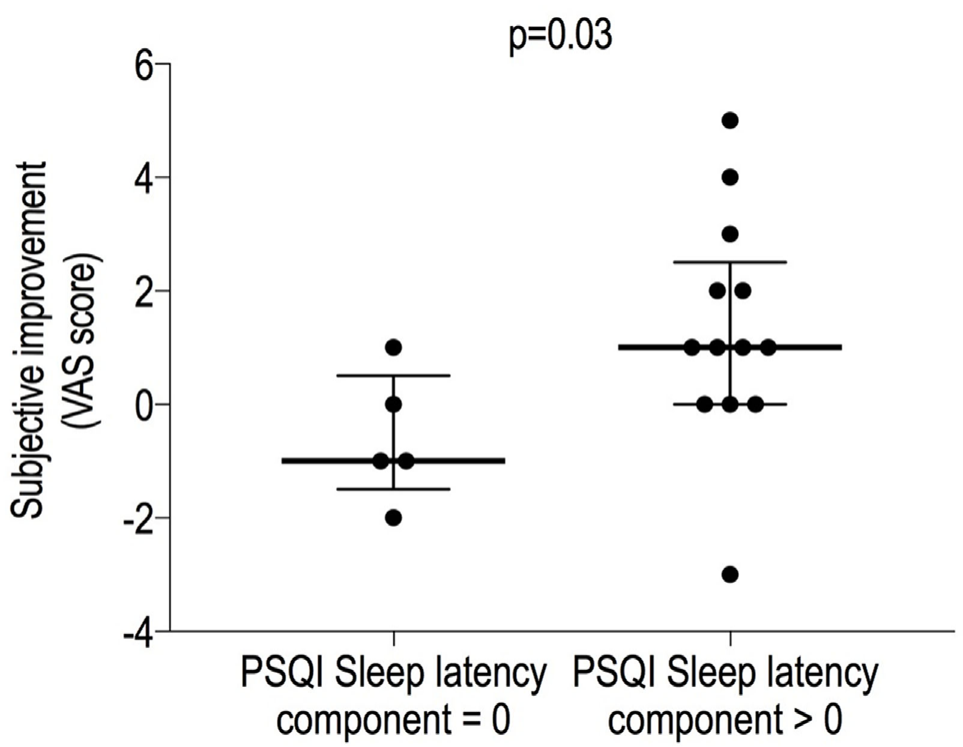
Clinical predictors of the subjective sleep quality improvement. The sleep latency component of the PSQI was found to be related to the VAS improvement: subjects who complained of insomnia were more likely to exhibit a better improvement in perceived sleep quality when broadband sound was administered. Data are expressed as median [interquartile range]. VAS, visual analog scale; PSQI, Pittsburgh Sleep Quality Index.

An interesting result of the current study is that the PSQI section 2 (sleep latency) score was the main predictor of the subjective improvement in sleep quality, meaning that subjects who complain of falling asleep slowly have a greater improvement in subjective sleep quality (VAS) compared with subjects who do not have difficulty with initiating sleep (*p* = 0.04, Figure 5). Hence, the PSQI section 2 might be used as a predictor for a better subjective response to filtered noise treatment.

### Methodological Considerations

This study has the following limitations. First, the sampled population consisted of young adults: since sleep architecture (41) and sleep quality perception (42) tend to change with age, older people might have had different responses to the filtered noise, even if the sleep onset has been demonstrated not to generally worsen with age (41). Second, subjects’ sleep initiation might have been affected by the equipment used (i.e., EEG electrodes, nasal cannula). However, the same equipment was used on the baseline night. Third, it must be emphasized that the subjects’ subjective sleep quality evaluation might be influenced by the single-blind study design, in which only the technician analyzing the sleep studies was blinded to the treatment allocation; a double-blind protocol would have been more desirable, although not compatible with the noise blanket night procedure. Fourth, subjective sleep onset was not recorded and thus it is unclear how this treatment might affect the patient’s perception of sleep onset. Nevertheless, we showed clear changes in objectively measured sleep latency. Fifth, we did not assess the impact of quieter or louder noise levels on sleep quality and architecture. However, this would have required much more data and ultimately we wanted to use a broadband sound level that was just above the measured ambient noise level of 40 dB. Sixth, we did not investigate psychological factors, e.g., traumatic events, that might impact sleep. However, complete histories were performed before each study and the participants did not disclose any such events. Seventh, time to bed prior the experiment was not standardized for the subjects. Nonetheless, all individuals were asked not to change their sleep schedules or habits during the study. Finally, the individuals studied in this protocol did not have a history of insomnia. However, we did use an accepted model of transient insomnia, in which subjects were put to bed 90 min earlier than their usual bedtime. Nevertheless, further study is needed to determine the effectiveness of this treatment in patients complaining of insomnia.

## Conclusion

In an experimental model of transient insomnia in young healthy individuals, broadband sound administration (Nightingale^®^ sound blanket) significantly reduced the latency to stable sleep by 38% when compared to normal environmental noise. Furthermore, subjects who complained about trouble initiating sleep (“slower sleepers”) showed subjective and objective sleep quality ameliorations with intervention. Our results suggest that these findings, if confirmed on insomnia patients, may lead the broadband sound administration to become a useful non-pharmacological tool to minimize sleep onset insomnia.

## Ethics Statement

The protocol was approved by the Partners Institutional Review Board at Brigham and Women’s Hospital. All subjects provided written informed consent before enrollment in the study. The trial was registered on http://www.clinicaltrials.gov (NCT02945254).

## Author Contributions

LM: contributed to data collection, data analysis and interpretation, and drafting and review of the manuscript for important intellectual content. LT-M: contributed to study design, data collection, data analysis and interpretation, and drafting and review of the manuscript for important intellectual content. SS: contributed to study design, data analysis, and review of the manuscript. MM: contributed to data collection and review of the manuscript. AA: contributed to data analysis and review of the manuscript. DAW: contributed to study design, final approval of the version submitted for publication, data analysis and interpretation, and review of the manuscript for important intellectual content.

## Conflict of Interest Statement

LT-M, SS, and AW received a consultancy fee from Cambridge Sound Management. LT-M received a consultancy fee from Novion Pharmaceuticals. AW has received a consultancy fee from Philips-Respironics, Bayer Pharmaceuticals. LM, AA, and MM have no conflicts of interest to disclose.
